# Ecologic Correlations of Selected Food Groups With Disease Incidence and Mortality in Switzerland

**DOI:** 10.2188/jea.JE20130029

**Published:** 2013-11-05

**Authors:** Harold Besson, Fred Paccaud, Pedro Marques-Vidal

**Affiliations:** Institute of Social and Preventive Medicine (IUMSP), Lausanne University Hospital, Lausanne, Switzerland

**Keywords:** ecologic correlation, food availability, standardized mortality rate, incidence, cardiovascular disease, cancer, Switzerland

## Abstract

**Background:**

There is little information regarding the impact of diet on disease incidence and mortality in Switzerland. We assessed ecologic correlations between food availability and disease.

**Methods:**

In this ecologic study for the period 1970–2009, food availability was measured using the food balance sheets of the Food and Agriculture Organization of the United Nations. Standardized mortality rates (SMRs) were obtained from the Swiss Federal Office of Statistics. Cancer incidence data were obtained from the World Health Organization Health For All database and the Vaud Cancer Registry. Associations between food availability and mortality/incidence were assessed at lags 0, 5, 10, and 15 years by multivariate regression adjusted for total caloric intake.

**Results:**

Alcoholic beverages and fruit availability were positively associated, and fish availability was inversely associated, with SMRs for cardiovascular diseases. Animal products, meat, and animal fats were positively associated with the SMR for ischemic heart disease only. For cancer, the results of analysis using SMRs and incidence rates were contradictory. Alcoholic beverages and fruits were positively associated with SMRs for all cancer but inversely associated with all-cancer incidence rates. Similar findings were obtained for all other foods except vegetables, which were weakly inversely associated with SMRs and incidence rates. Use of a 15-year lag reversed the associations with animal and vegetal products, weakened the association with alcohol and fruits, and strengthened the association with fish.

**Conclusions:**

Ecologic associations between food availability and disease vary considerably on the basis of whether mortality or incidence rates are used in the analysis. Great care is thus necessary when interpreting our results.

## INTRODUCTION

Food availability influences health. Some foods are considered risk factors for selected cardiovascular diseases^1^^,^^2^ and cancers.^3^ Several studies have shown a link between food availability and mortality, namely in the transition from animal to vegetable fats (saturated to unsaturated fats) and the consumption of fruits and vegetables,^4^ fish,^5^ sugar, and salt.^6^ However, the underlying mechanisms linking food availability with diseases remain to be investigated.^7^

Switzerland is a small European country that has witnessed a marked decrease in mortality rates during the last 20 years.^8^ Significant changes in food availability have also occurred: fat and sugar intakes increased, while intakes of total carbohydrate, fruits, and vegetables decreased.^9^ In 2007, protein, fat, carbohydrates, and alcohol represented 10.8%, 40.3%, 43.7%, and 5.2%, respectively, of the total caloric supply.^9^ A study of trends in the dietary intake of the population of Geneva between 1999 and 2009 showed no change in total energy intake, although intakes of calcium, iron, and polyunsaturated fatty acids significantly decreased.^10^ It is unclear whether the decrease in mortality rates in Switzerland is partly attributable to changes in food availability. With a few exceptions,^11^^,^^12^ there are no data on individual dietary intake in Switzerland; hence, we assessed the ecologic correlations between food availability and mortality/incidence of cardiovascular and cancer diseases during the period 1970–2009. We also investigated whether the results were similar in separate analyses of mortality and incidence data.

## METHODS

### Food availability

Food availability was obtained from food balance sheets produced by the Food and Agriculture Organization of the United Nations (FAO).^13^ The food balance sheets estimate the availability of selected foods for a given country by combining domestic production, imports and exports, stocks, and non-food use. The resulting yearly supply of each food is then divided by the average population and the number of days of the corresponding year to obtain the individual daily availability of each food commodity. For this study, we used the corresponding calories of each food commodity (kcal/person/day) to assess ecologic correlations. The following food commodities were selected from the FAO database: total energy, all animal products, all vegetal products, cereals (wheat, maize, barley, other; excluding beer), sugars and sweeteners (sugar, honey), vegetable oils (olive, groundnut, other), alcoholic beverages (wine, beer, other fermented drinks), meat (beef, pork, poultry, other), and milk (any type; excluding butter). Food commodities representing at least 10% of total caloric intake were selected. In addition, among foods representing less than 10% of total caloric intake, fish (pelagic, demersal, seafood), fruits (apples, oranges, other), vegetables (eg, carrots, spinach), and animal fats (butter, gee, other) were selected, as they are classically associated with disease. For more information, consult http://faostat.fao.org/site/368/default.aspx#ancor.

### Mortality data

Standardized mortality rates (SMR) per 100 000 inhabitants for each year during the period 1970–2009 were obtained from the Federal Office of Public Health for the following diseases (International Statistical Classification of Diseases, 10th Revision [ICD-10] code): circulatory system (I00–I99), ischemic heart disease (I20–I25), cerebrovascular diseases (I60–I69), malignant neoplasms (C00–C97), cancer of the trachea/bronchus/lung (C33–C34), cancer of the cervix (C53), and female breast cancer (C50). No national data on colorectal cancer mortality were available. Rates were adjusted by direct standardization according to the European standard population.

### Incidence data

Incidence rates for the period 1980–2008 were obtained from the World Health Organization Health for All Database^14^ for the following types of cancer: all, cervix, trachea/bronchus/lung, and female breast. Incidence rates for colorectal cancer and polyps for the period 1983–2007 were obtained from the Vaud Cancer Registry.^15^

### Statistical analysis

Statistical analyses were performed using Stata version 12.0 (Stata Corp, College Station, TX, USA). Ecologic correlations between total energy and mortality/incidence were assessed using Spearman rank correlation coefficients. For all other food commodities, a multivariate regression analysis adjusting for total caloric intake was conducted as previously described,^16^ and the results were expressed as standardized coefficients. Standardized coefficients are unit-independent and can be interpreted in the same way as correlation coefficients. Because food availability might exert effects after a time lag in cancer, an analysis was conducted using lags of 0, 5, 10, and 15 years as in previous research.^16^^,^^17^ For example, an analysis at lag 5 assesses the association between food availability at a given time (eg, 1975) and mortality/incidence 5 years later (ie, 1980). For this analysis, only coefficients exceeding the cut-off of ±0.70 were considered meaningful.

## RESULTS

### Cardiovascular diseases

The ecologic correlations between food availability and the SMRs for total mortality and cardiovascular disease mortality are summarized in Table 1. Alcoholic beverages and fruit availability were positively associated, while fish availability was inversely associated, with SMRs for almost all types of cardiovascular disease. Animal products, meat, and animal fats were positively associated with the SMR for ischemic heart disease only.

**Table 1.** Ecologic correlations between secular trends in the availability of selected food commodities and European-standardized mortality rates of all-cause death and death due to cardiovascular diseases for each year during the period 1970–2009, Switzerland

	Total mortality	Diseases of the circulatory system	Ischemic heart disease	Cerebrovascular diseases
Total energy	0.265	0.254	0.193	0.266
Animal products				
All	0.388	0.468	0.730	0.373
Meat	0.357	0.431	0.707	0.335
Fish	−0.905	−0.908	−0.681	−0.932
Milk	0.302	0.397	0.534	0.329
Animal fats	0.567	0.606	0.754	0.543
Vegetal products				
All	−0.479	−0.578	−0.902	−0.460
Cereals	0.074	−0.001	−0.127	0.041
Sugar/sweeteners	−0.762	−0.812	−0.952	−0.737
Vegetable oils	−0.429	−0.479	−0.781	−0.374
Fruits	0.855	0.874	0.889	0.834
Vegetables	−0.519	−0.470	−0.125	−0.521
Alcoholic beverages	0.815	0.838	0.789	0.801

Results are expressed as Spearman nonparametric correlations for total energy and as standardized regression coefficients adjusted for total energy for the other food commodities. Coefficients exceeding the cut-off of ±0.70 are considered meaningful.

### Cancer

The ecologic correlations between food availability and incidence rates and SMRs for cancer are summarized in Table 2. The results for SMRs and incidence rates were contradictory. Alcoholic beverages and fruits were positively associated with all-cancer SMRs but inversely associated with all-cancer incidence rates (Table 2). Similar findings were obtained for all other foods except vegetables, which were weakly inversely associated with SMRs and incidence rates (Table 2). Similarly contradictory results were also observed for cancers of the trachea/bronchus/lung and breast (Table 2). The incidence trends for colorectal polyps were very similar to those for all cancer, while colorectal cancer was not strongly associated with any food.

**Table 2.** Ecologic correlations of secular trends in the availability of selected food commodities with cancer incidence and mortality rates; 1970–2009, Switzerland

	All cancers		Trachea/bronchus/lung		Cervix		Breast	
				
	Mortality	Incidence	Mortality	Incidence	Mortality	Incidence	Mortality	Incidence
Total energy	0.277	−0.147	−0.024	0.217	0.284	0.086	0.068	−0.165
Animal products								
All	0.688	−0.974	0.946	−0.377	0.376	0.935	0.796	−0.975
Meat	0.673	−0.950	0.952	−0.310	0.345	0.892	0.784	−0.959
Fish	−0.733	0.836	−0.290	0.522	−0.889	−0.798	−0.571	0.783
Milk	0.520	−0.842	0.760	−0.357	0.310	0.800	0.604	−0.851
Animal fats	0.666	−0.764	0.577	−0.563	0.526	0.830	0.678	−0.699
Vegetal products								
All	−0.850	0.947	−0.924	0.368	−0.464	−0.910	−0.983	0.949
Cereals	−0.172	0.418	−0.573	−0.122	0.015	−0.426	−0.299	0.486
Sugar/sweeteners	−0.931	0.927	−0.890	0.586	−0.730	−0.866	−0.948	0.877
Vegetable oils	−0.698	0.879	−0.844	0.334	−0.372	−0.866	−0.782	0.855
Fruits	0.885	−0.903	0.646	−0.509	0.829	0.887	0.854	−0.864
Vegetables	−0.341	−0.179	0.105	−0.377	−0.562	0.333	−0.224	−0.047
Alcoholic beverages	0.911	−0.931	0.752	−0.303	0.794	0.862	0.875	−0.916

Results are expressed as Spearman nonparametric correlations for total energy and as standardized regression coefficients adjusted for total energy for the other food commodities. Coefficients exceeding the cut-off of ±0.70 are considered meaningful.

Assessment of SMR and incidence rates over time showed divergent trends, namely, a decrease in SMRs and an increase in incidence rates (Figure).

**Figure.**
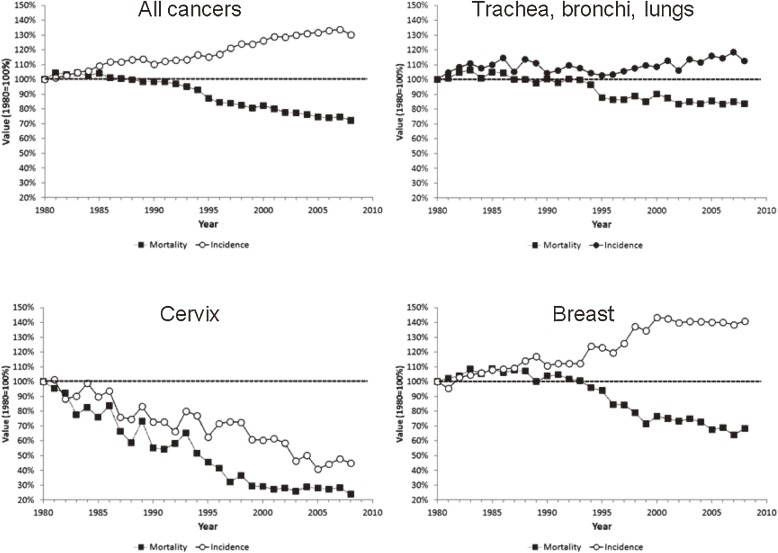
Trends in incidence (open circles) and standardized mortality ratios (closed squares) for cancer (Switzerland, 1980–2009). Values are reported using 1980 as a reference (100%).

### Effect of lag time

The results of analyses of lag time between food availability and cancer incidence rates are summarized in Tables 3A and 3B. For all-cancer incidence and mortality, the associations with all vegetal products, animal products, vegetable oils, and meat tended to reverse as the lag increased. Conversely, the strength of associations with fish availability tended to increase. Most associations of food availability with trachea/bronchus/lung and cervix cancer decreased with increasing lag. Finally, the associations of alcohol and fruit availability with cancer incidence rates remained relatively stable and tended to weaken after 15 years.

**Table 3A.** Effect of lag time on the association of the availability of selected food commodities with all-cancer and trachea/bronchus/lung cancer incidence rates; 1970–2009, Switzerland

	Lag time (years)							
	
	All cancers				Cancer of the trachea/bronchus/lung			
		
	0	5	10	15	0	5	10	15
Total energy	−0.147	−0.231	−0.662	−0.412	0.217	0.337	−0.570	−0.157
Animal products								
All	−0.974	−0.895	0.084	0.448	−0.377	−0.730	−0.284	−0.297
Meat	−0.950	−0.814	0.124	0.559	−0.310	−0.730	−0.264	−0.201
Fish	0.836	0.793	0.748	0.939	0.522	0.348	−0.016	0.375
Milk	−0.842	−0.885	−0.057	0.059	−0.357	−0.573	−0.212	−0.339
Animal fats	−0.764	−0.505	−0.157	−0.155	−0.563	−0.255	−0.194	−0.616
Vegetal products								
All	0.947	0.807	−0.109	−0.693	0.368	0.656	0.368	0.460
Cereals	0.418	0.462	−0.029	−0.425	−0.122	0.361	0.349	0.259
Sugar/sweeteners	0.927	0.851	0.250	0.153	0.586	0.638	0.198	0.637
Vegetable oils	0.879	0.477	−0.196	−0.605	0.334	0.482	0.250	0.074
Fruits	−0.903	−0.837	−0.619	−0.644	−0.509	−0.580	−0.178	−0.312
Vegetables	−0.179	0.742	0.491	0.485	−0.377	0.722	0.138	−0.127
Alcoholic beverages	−0.931	−0.784	−0.654	−0.361	−0.303	−0.529	−0.476	−0.426

Results are expressed as Spearman nonparametric correlations for total energy and as standardized regression coefficients adjusted for total energy for the other food commodities. Coefficients exceeding the cut-off of ±0.70 are considered meaningful.

**Table 3B.** Effect of lag time on the association of the availability of selected food commodities with incidence rates for cervical and breast cancer; 1970–2009, Switzerland

	Lag time (years)							
	
	Cervical cancer				Breast cancer			
		
	0	5	10	15	0	5	10	15
Total energy	0.086	0.192	0.495	0.442	−0.165	−0.314	−0.605	−0.455
Animal products								
All	0.935	0.847	−0.101	−0.377	−0.975	−0.861	0.107	0.575
Meat	0.892	0.758	−0.130	−0.472	−0.959	−0.787	0.164	0.678
Fish	−0.798	−0.845	−0.771	−0.828	0.783	0.745	0.797	0.924
Milk	0.800	0.821	0.023	−0.111	−0.851	−0.811	−0.070	0.137
Animal fats	0.830	0.611	0.112	0.214	−0.699	−0.518	−0.210	0.001
Vegetal products								
All	−0.910	−0.764	0.130	0.584	0.949	0.776	−0.139	−0.891
Cereals	−0.426	−0.429	0.019	0.259	0.486	0.456	−0.034	−0.655
Sugar/sweeteners	−0.866	−0.827	−0.213	−0.010	0.877	0.819	0.226	0.118
Vegetable oils	−0.866	−0.464	0.206	0.431	0.855	0.454	−0.219	−0.678
Fruits	0.887	0.821	0.654	0.652	−0.864	−0.772	−0.637	−0.552
Vegetables	0.333	−0.644	−0.574	−0.452	−0.047	0.657	0.450	0.558
Alcoholic beverages	0.862	0.747	0.729	0.519	−0.916	−0.812	−0.626	−0.245

Results are expressed as Spearman nonparametric correlations for total energy and as standardized regression coefficients adjusted for total energy for the other food commodities. Coefficients exceeding the cut-off of ±0.70 are considered meaningful.

The results regarding the incidence of colorectal polyps and cancer are summarized in Table 4. Several associations with correlation coefficients greater than the ±0.70 threshold were found for polyps, but no corresponding associations were found for colorectal cancer incidence. Finally, some associations between food availability and incidence of polyps strengthened with increasing lag time (eg, fish), while others decreased (eg, animal fats).

**Table 4.** Effect of lag time on the association of the availability of selected food commodities with incidence rates for colorectal polyps and colorectal cancer; 1970–2009, Switzerland

	Lag time (years)							
	
	Polyps				Colorectal cancer			
		
	0	5	10	15	0	5	10	15
Total energy	0.069	−0.525	−0.513	−0.457	−0.078	−0.175	0.091	0.067
Animal products								
All	−0.858	−0.905	−0.435	0.316	−0.052	−0.054	0.056	0.351
Meat	−0.844	−1.000	−0.401	0.415	−0.064	−0.075	0.072	0.381
Fish	0.486	0.573	0.603	0.855	0.037	−0.284	0.118	0.272
Milk	−0.712	−0.865	−0.477	0.010	−0.115	0.062	0.060	0.137
Animal fats	−0.696	−0.494	−0.233	−0.245	0.116	−0.055	−0.127	0.187
Vegetal products								
All	0.919	0.827	0.478	−0.493	0.056	0.043	−0.063	−0.547
Cereals	0.494	0.536	0.298	−0.144	0.232	0.180	0.287	−0.680
Sugar/sweeteners	0.979	0.736	0.571	0.075	−0.068	−0.076	−0.348	0.098
Vegetable oils	0.881	0.728	0.079	−0.461	−0.114	0.079	−0.059	−0.218
Fruits	−0.911	−0.825	−0.676	−0.669	−0.018	0.224	−0.093	0.086
Vegetables	−0.209	0.550	0.384	0.376	0.515	−0.265	−0.112	0.060
Alcoholic beverages	−0.747	−0.817	−0.755	−0.491	0.142	−0.133	0.051	0.085

Results are expressed as Spearman nonparametric correlations for total energy and as standardized regression coefficients adjusted for total energy for the other food commodities. Coefficients exceeding the cut-off of ±0.70 are considered meaningful.

## DISCUSSION

Ecologic studies can provide important information for public health interventions.^18^ Ecologic correlations are based on aggregated data such as mortality rates or number of doctors per 10 000 inhabitants. Analyses usually rely on simple association statistics such as correlation coefficients and linear regression estimates between a variable of interest (eg, mortality rates) and putative determinants such as the number of doctors per 10 000 inhabitants, diet, or socioeconomic markers such as mean income or even refrigerator use.^19^ Ecologic correlations can use data from different countries,^20^^,^^21^ different regions within a single country,^22^ or different time points within a country.^4^ This methodology has been used to assess associations between diet and disease in Poland,^4^ Spain,^23^ Finland,^24^ Japan,^25^ Korea,^17^ and in several countries simultaneously.^20^^,^^21^ In this study, we used the second method to assess the association between food availability and disease in Switzerland. To our knowledge, this is the first study of this kind in Switzerland. Our results suggest that associations between food availability and disease vary considerably according to the disease parameter (SMR or incidence rate) and time lag considered. Hence, great care should be taken when interpreting the results.

### Cardiovascular disease

Animal products (including meat and fats) were positively associated, while fish availability was negatively associated, with the SMR for ischemic heart disease. Similar findings were reported in some^4^^,^^20^ but not all^26^ ecologic studies. Possible explanations are the high content of saturated fatty acids in animal products and the high content of polyunsaturated fatty acids in fish, both of which were found to modulate CVD in prospective studies.^27^ We noted a small inverse association between vegetable availability and the SMRs for cardiovascular diseases, which is also in agreement with the literature.^28^ In contrast, an example of the ecologic fallacy was observed in the association between fruit availability and the SMRs for all CVDs.

### Cancer

Alcoholic beverage availability was positively associated with cancer mortality, as was the case in previous studies.^29^ Similarly, the inverse association between fish availability and cancer mortality is in agreement with the literature.^3^ However, the positive association between fruit availability and cancer mortality contradicts previous findings.^30^ Finally, the association of sugar/sweeteners with cancer mortality might be mediated by obesity and diabetes.^31^

Food availability was more strongly associated with cancer incidence than with cancer mortality. In addition, some associations contradicted the findings of previous studies.^32^ Our findings that animal products and meat availability were inversely associated with cancer incidence contradict previous research.^32^^–^^34^ The inverse association between fruit intake and cancer incidence, possibly due to the protective effect of increased fiber intake,^30^ agreed with previous findings.^19^^,^^35^ The inverse association between alcoholic beverage availability and cancer incidence might be attributable to the protective effect of moderate drinking,^36^ although this favorable association has been recently challenged.^37^

Overall, our results indicate that ecologic associations between food availability and cancer vary (and even reverse) depending on whether mortality or incidence rates are used. Hence, the impact of dietary changes on cancer cannot be adequately estimated using ecologic correlations, and recommendations regarding dietary prevention should not be based solely on such studies. Our results indicate that ecologic associations might lead to erroneous findings (ecologic fallacy) and that recommendations should be based on results from prospective and intervention studies.

Although most studies found no association between diet and cancer of the respiratory tract,^38^^,^^39^ we observed strong correlations between food availability and mortality from cancer of the trachea, bronchus, and lungs. Although a positive association between meat availability and lung cancer mortality has been reported,^40^ the most likely explanation is that cancer rates and diet evolved separately, and that the association is purely incidental. Indeed, when associations of food availability with incidence and mortality from several types of cancer were assessed, the findings were contradictory. Fruit availability was positively associated with cervical cancer mortality, which contradicts the results of prospective studies^41^ and evidence cited in reviews of existing evidence,^32^ again suggesting an ecologic fallacy. We found that alcoholic beverages were positively associated with breast cancer mortality, a consistent and plausible finding that was also reported previously.^32^^,^^40^ In contrast, alcoholic beverages and fruit availability were inversely associated with breast cancer incidence, a finding that contradicts previous results.^32^ The positive associations of vegetal products, vegetable oils, and fish with breast cancer incidence also contradicts the results of prospective studies,^42^^,^^43^ and a recent review found no evidence of an association between these foods and breast cancer.^32^ Nevertheless, some of the observed associations are metabolically plausible. Low fruit consumption might increase breast cancer risk, because of low fiber intake,^44^ although other studies did not identify such an association.^42^ Similarly, sugar and sweeteners could increase breast cancer incidence via increased obesity and diabetes.^45^

The pathogenesis and development of colorectal cancer are influenced by a variety of foods.^32^^,^^46^^,^^47^ Hence, we assessed associations between dietary availability and incidence of colorectal polyps (a precancerous condition) and cancer, using data from the Vaud Cancer Registry. No clear association was found between food availability and colorectal cancer incidence, a finding that contradicts the results of other studies.^32^ The positive association of sugar and sweetener availability with colorectal polyp incidence might partly be explained by increased obesity levels,^48^ while the inverse association between fruit availability and colorectal polyp incidence is in agreement with the literature.^32^^,^^49^ Conversely, vegetal products and vegetable oils were positively associated with colorectal polyp incidence, while animal products, milk, meat, and fat availability were inversely associated with colorectal polyp incidence. These findings contradict those of previous studies.^32^^,^^33^^,^^50^^,^^51^

Thus, our findings suggest that the results of cross-sectional ecologic correlations regarding cancer should be evaluated with considerable caution, as they vary considerably depending on whether mortality or incidence rates are used.

### Effect of lag time

Time-trend ecologic studies are prone to temporal ambiguity due to the latent period between exposure and disease occurrence.^18^ Breast cancer may take 1 to 6 years to develop, and colorectal cancer may require 6 to 16 years.^52^ To address this effect, correlations between food availability and cancer incidence were assessed at different time lags, as in previous studies.^16^^–^^18^^,^^40^ Our results show that different patterns are possible. For instance, the “paradoxical” associations (ie, those not in agreement with the literature) of vegetal products, animal products, vegetable oils, and meat availability with cancer incidence reversed after 15 years, at which point our findings agreed with those of previous studies.^17^^,^^33^^,^^34^^,^^41^ This change in the direction of the association between diet and cancer was reported previously.^40^^,^^53^ Still, we were unable to define a precise lag time. The lag corresponding to the strongest correlation between meat and breast cancer incidence was 15 years in the present study and 10 years in studies conducted in Korea^17^ or Hong Kong.^40^

The associations of alcohol and fruit availability with cancer incidence decreased with increasing lag time, but no change in the direction of the associations was seen. Finally, the strength of the association between fish availability and cancer incidence increased with increasing lag time. Overall, our results suggest that the direction and strength of the association between diet and cancer incidence vary not only according to lag time but also according to food type. Hence, we must again emphasize that great caution is required in interpreting ecologic correlations.

### Study strengths and limitations

Ecologic studies have several advantages. They are inexpensive, easy to perform, and can be used when individual data are missing. When adequately interpreted, these studies can provide interesting information for public health interventions.^18^ The ever-increasing availability of online data further facilitates such studies.

However, ecologic studies have several limitations. First, they rely on aggregate data, and dietary intake varies considerably within and between subjects.^54^ Second, food availability is limited to certain macronutrients (protein, fat, and carbohydrates), and no data on micronutrients (vitamins and minerals) are available. Third, the results of an ecologic correlation study cannot be directly applied to individuals^55^; although dietary changes might benefit a whole population,^1^ this effect could miss the groups at higher risk, such those with lower incomes. This phenomenon is known as the ecologic fallacy.^18^ Fourth, it is likely that the diverging trends for cancer incidence and mortality were due to improvements in diagnostic capacity and treatment; however, we were unable to account for such changes. Fifth, it was not possible to adjust for other confounding factors such as smoking and obesity levels, as these data were not continuously collected in Switzerland. Finally, association is not causation, and other factors such as improvements in cancer treatment may have caused the changes in mortality, which would decrease mortality even though a greater number of cancers are diagnosed.^56^ Only prospective studies of the association between individual dietary intake and disease incidence will enable estimation of the impact of diet on cardiovascular disease and cancer.

We conclude that in Switzerland ecologic associations between food availability and disease vary considerably depending on whether mortality or incidence rates are used in the analysis. Hence, caution is required in interpreting the results.
